# Meteorological Table, for the Month of December, 1851

**Published:** 1852-01

**Authors:** 


### METEOROLOGICAL TABLE,
### FOR THE MONTH OF DECEMBER, 1851.

| Day of month, | Morning, | 2 o'clock, P.M., | Evening, | Mean temperature, | Barometer, | Rain-guage, | Course of wind, | REMARKS. |
|---|---|---|---|---|---|---|---|---|
| 1 | 29 | 47 | 35 | 37 | 29.15 | | e. | Variable. |
| 2 | 38 | 41 | 40 | 40 | 29.38 | 08 | w. | Cloudy. |
| 3 | 31 | 33 | 32 | 32 | 29.38 | | n. | Do. |
| 4 | 31 | 36 | 34 | 34 | 29.47 | | n. w. | Do. |
| 5 | 29 | 36 | 30 | 32 | 29.55 | | e. n. e. | Variable. |
| 6 | 20 | 49 | 39 | 36 | 29.56 | | s. s. w. | Do. |
| 7 | 39 | 58 | 47 | 48 | 29.53 | | s. w. | Do. |
| 8 | 52 | 50 | 47 | 50 | 29.37 | 06 | n. w. | Cloudy. |
| 9 | 32 | 39 | 36 | 36 | 29.55 | 04 | calm. | Do. |
| 10 | 35 | 37 | 35 | 36 | 29.19 | 46 | n. n. w. | Do. |
| 11 | 30 | 32 | 31 | 31 | 29.30 | | n. w. | Do. |
| 12 | 30 | 35 | 35 | 33 | 29.10 | | w. | Do. |
| 13 | 12 | 21 | 17 | 17 | 29.19 | | w. s. w. | Clear. |
| 14 | 22 | 39 | 41 | 34 | 29.02 | 05 | s. s. w. | Cloudy. |
| 15 | 8 | 11 | 5 | 8 | 29.17 | 16 | n. w. | Variable. |
| 16 | 5* | 11 | 8 | 5 | 29.34 | | w. n. w. | Do. |
| 17 | 5* | 11 | 11 | 6 | 29.31 | | swly. | Clear. |
| 18 | 11 | 20 | 16 | 16 | 29.05 | | w. s. w. | Variable. |
| 19 | 12 | 33 | 33 | 26 | 29.08 | | calm. | Clear. |
| 20 | 19 | 28 | 23 | 23 | 29.18 | | n. n. w. | Variable. |
| 21 | 20 | 24 | 22 | 32 | 29.18 | | n. e. | Cloudy. |
| 22 | 22 | 29 | 26 | 26 | 29.24 | 20 | s. w. | Do. |
| 23 | 4* | 24 | 10 | 10 | 29.50 | | calm. | Clear. |
| 24 | 18 | 43 | 36 | 32 | 29.24 | | n. n. w. | Variable. |
| 25 | 35 | 42 | 40 | 39 | 29.19 | 20 | s. w. | Cloudy. |
| 26 | 39 | 30 | 30 | 33 | 29.39 | 06 | s. w. | Do. |
| 27 | 37 | 53 | 51 | 47 | 29.16 | 10 | s. s. w. | Do. |
| 28 | 50 | 55 | 50 | 52 | 28.95 | 46 | calm. | Variable. |
| 29 | 40 | 64 | 61 | 55 | 29.10 | | s. s. w. | Do. |
| 30 | 55 | 48 | 41 | 48 | 29.07 | 1.40 | n. n. w. | Cloudy. |
| 31 | 34 | 38 | 37 | 36 | 39.11 | 64 | n. n. w. | Do. |
| | | | | 31.6 | | 3.91 | | |

*Below zero.

